# BKI-1748 confers a high level of protection against ovine congenital toxoplasmosis when administered after IgM seroconversion

**DOI:** 10.3389/fcimb.2026.1819490

**Published:** 2026-04-27

**Authors:** Roberto Sánchez-Sánchez, Natalia Velasco-Jiménez, David Arranz-Solís, Miguel Criado, Ignacio Ferre, Michela Re, Matthew A. Hulverson, Ryan Choi, Lynn K. Barrett, Andrew Hemphill, Wesley C. Van Voorhis, Luis Miguel Ortega-Mora

**Affiliations:** 1 SALUVET, Animal Health Department, Faculty of Veterinary Sciences, Complutense University of Madrid, Ciudad Universitaria s/n, Madrid, Spain; 2 Instituto de Ganadería de Montaña, (CSIC-ULE), Grulleros, León, Spain; 3 Animal Medicine and Surgery Department, Faculty of Veterinary Sciences, Complutense University of Madrid, Madrid, Spain; 4 Center for Emerging and Re-emerging Infectious Diseases (CERID), Division of Allergy and Infectious Diseases, Department of Medicine, University of Washington, Seattle, WA, United States; 5 Institute of Parasitology, Vetsuisse Faculty, University of Berne, Berne, Switzerland

**Keywords:** BKI-1748, congenital toxoplasmosis, efficacy, IgM, sheep, *Toxoplasma gondii*, translational pharmaceutics

## Abstract

**Background:**

Unlike mouse models of congenital toxoplasmosis, pregnant sheep models provide the opportunity to evaluate treatment strategies that more closely resemble clinical practice in pregnant women, including chemotherapeutic interventions initiated after specific IgM seroconversion. BKI-1748, which targets *Toxoplasma gondii* CDPK1 and MAPKL-1 protein kinases, has demonstrated an excellent safety profile and efficacy when administered repeatedly to pregnant sheep, starting at 2 and 7 days after challenge.

**Methods:**

In this study, treatment was initiated at day 14 post-infection (p.i.), following *T. gondii* IgM seroconversion. Twenty-three sheep were orally inoculated with 10 TgShSp1 oocysts at 90 days of gestation, while three sheep remained uninfected. On day 14 p.i., infected sheep carrying live fetuses (n = 10) received 10 doses of BKI-1748 orally at 15 mg/kg every 2 days, whereas 10 infected sheep were left untreated.

**Results:**

All infected sheep, both treated and untreated, seroconverted to serum IgG by day 21 p.i., with treated sheep showing a marked reduction in IgG levels from day 28 p.i. onward. Administration of the compound significantly enhanced lamb viability in infected sheep, resulting in 91% viable lambs in treated animals compared to 52% in untreated sheep. Whereas all lambs born to untreated sheep were congenitally infected, only 17% of lambs in the treated group were infected. Nevertheless, congenitally infected lambs in the treated group had lower birth weights than *T. gondii*-free lambs.

**Conclusion:**

This study highlights the potential utility of BKI-1748 for prenatal treatment of human congenital toxoplasmosis, in which IgM seroconversion prompts the need for intervention.

## 1 Introduction

Toxoplasmosis is a zoonotic disease caused by the apicomplexan parasite *Toxoplasma gondii*, infecting approximately one third of the global population and typically resulting in a subclinical infection in immunocompetent individuals. Humans, as well as all homeothermic vertebrates, can become infected through the ingestion of sporulated oocysts in contaminated water or vegetables (infection route in sheep) or tissue cysts present in infected meat (Dubey, 2021). Congenital toxoplasmosis occurs when maternal infection is acquired for the first time in pregnancy, allowing *T. gondii* to cross the placenta and enter the fetal circulation during the phase of parasitemia. Congenital *T. gondii* infections in humans occur at an estimated incidence of approximately 0.5 cases per 1,000 live births in North America and Western Europe, and 1.8–3.4 cases per 1,000 live births in low-income countries in Central America, South America, and Africa, and are of major public health concern due to the severe clinical consequences for newborns (Torgerson and Mastroiacovo, 2013). Congenital toxoplasmosis can lead to fetal death, abortion, and syndromes that affect neurological function, cognitive abilities, and the retina (Maldonado and Read, 2017; Bollani et al., 2022; Garweg et al., 2022). Infections with *T. gondii* during early pregnancy generally results in more severe fetal outcomes, although vertical transmission is more frequently observed in the latter half of gestation (Dubey et al., 2021). In small ruminants, *Toxoplasma gondii* accounts for 10-23% of ovine abortions in Europe and the USA, and likely a higher proportion in the Middle East and South America (Stelzer et al., 2019), resulting in economic losses of €63 and €171 per abortion in meat and dairy flocks, respectively (Gutiérrez-Expósito et al., 2021). The control of this zoonotic disease requires joint efforts from both the human and veterinary medical perspectives, in line with the One Health approach (Aguirre et al., 2019). In sheep, an attenuated live vaccine (Toxovax™) is licensed in some countries and reduces lamb losses and congenital infection by approximately two-thirds (Buxton et al., 1991). In humans, the control measures to be applied include prevention, diagnosis, and treatment.

In humans, the screening strategy adopted by different countries for the diagnosis of congenital toxoplasmosis (prenatal, postnatal, or no systematic screening) depends on the incidence of the disease (Paquet et al., 2013; Wallon et al., 2014; Binquet et al., 2019). In pregnant women, determining the timing of infection is challenging, and serological techniques are the only tool to determine whether hosts acquired an acute infection during gestation (Konstantinovic et al., 2019; Fricker-Hidalgo et al., 2020). IgM antibodies are commonly used for early diagnosis, and IgM seroconversion (IgG-negative at the first positive IgM test) typically occurs between one and two weeks post-infection (p.i.) (Fricker-Hidalgo et al., 2020). Prenatal treatment with folate inhibitors that cross the placental barrier is often initiated after IgM seroconversion when fetal infection is strongly suspected or approximately one month after infection, following detection of the parasite in amniotic fluid (Marques et al., 2012; Paquet et al., 2013; Villard et al., 2016). However, if drug administration is delayed beyond one month after maternal infection, its efficacy decreases. Some studies report evidence suggesting that the odds of maternal–child transmission and clinical manifestations in the children may be lower when treatment is started within three weeks of maternal IgM seroconversion, compared with initiation at eight weeks (Gilbert and Peckham, 2002; Thiébaut et al., 2007; Mandelbrot et al., 2018).

Therapeutic schemes for congenital infections in humans have barely evolved for many years, with folate inhibitors (for confirmed fetal infections) and spiramycin (for fetal prophylaxis) being prescribed (Montoya and Rosso, 2005; Mandelbrot, 2020). Furthermore, there are considerable discrepancies regarding the efficacy of prenatal treatment in reducing the incidence and severity of congenital toxoplasmosis, as several studies reported contradictory and potentially biased results (Robert-Gangneux, 2014; Montoya, 2018; Konstantinovic et al., 2019; Bollani et al., 2022). Regarding the mechanism of action and adverse effects of folate inhibitors, pyrimethamine is a dihydrofolate reductase (DHFR) inhibitor that decreases active folate levels in humans and can cause teratogenic effects if administered during the first trimester of pregnancy, including neural tube defects. Sulfadiazine, administered synergistically with pyrimethamine, inhibits dihydropteroate synthase (DHPS) in the parasite, blocking folate synthesis and consequently impairing DNA synthesis; however, its use is limited by allergic dermatologic reactions and gastrointestinal adverse effects. To reduce the toxicity of folate inhibitors in humans, they are usually applied in combination with leucovorin (folinic acid), which can be taken up by human cells but not by *T. gondii* that lacks the corresponding transporter (Ben-Harari et al., 2017; Shammaa et al., 2021). In small ruminants, decoquinate is licensed in the European Union for toxoplasmosis, although the efficacy in reducing abortions is limited to 22%, even when administered in feed prior to infection (Buxton et al., 1996; Sanchez-Sanchez et al., 2018).

In recent decades, considerable progress has been made in the development of parasite-targeted therapies, including the investigation and repurposing of existing drugs (Neville et al., 2015; Alday and Doggett, 2017; Dunay et al., 2018; Elsheikha et al., 2020). Calcium dependent protein kinase 1 (CDPK1) is essentially involved in signaling processes that lead to *T. gondii* host cell invasion and egress and has no homologs in mammalian cells (Kieschnick et al., 2001; Lourido et al., 2010; Murphy et al., 2010; Ojo et al., 2010; Lourido et al., 2012; Cardew et al., 2018). TgCDPK1 can be selectively targeted by a class of ATP-competitive compounds named bumped kinase inhibitors (BKIs) (Van Voorhis et al., 2017). Recent evidence suggests that BKIs also inhibit *T. gondii* by targeting TgMAPKL-1, an essential cell-cycle regulatory kinase (Montgomery et al., 2025). BKI compounds based on the 5-aminopyrazole-4-carboxamide (AC) scaffold, such as BKI-1748, have demonstrated an adequate safety window *in vitro*, in zebrafish embryos, and in pregnant mice (Anghel et al., 2020; Imhof et al., 2021). However, affinity chromatography assessments identified some BKI-1748-binding proteins from zebrafish embryos involved in translation and RNA processing, suggesting potential off-target activity of the compound (Müller et al., 2022). In sheep, BKI-1748 has been shown to be safe when administered at 15 mg/kg every two days for ten doses during mid-pregnancy (Sánchez-Sánchez et al., 2024). In an effort to assess human-specific safety, BKI-1748 exhibited minimal inhibition of the hERG (human ether-à-go-go-related gene) potassium channel, which plays a critical role in regulating action potentials in cardiac myocytes, thereby demonstrating acceptable cardiovascular safety in dogs and rats (Hulverson et al., 2019; Choi et al., 2020). Regarding efficacy, administration of BKI-1748 starting at 2 days p.i. (when dissemination of *T. gondii* tachyzoites is unlikely to be extensive and infection remains undetected) was highly effective against congenital toxoplasmosis in both pregnant mice and pregnant sheep (Imhof et al., 2021; Sánchez-Sánchez et al., 2024). Furthermore, BKI-1748 was also highly effective in reducing lamb mortality and preventing vertical transmission of infection when administered in sheep from day 7 p.i. onwards (coinciding with the peak of fever and increased serum IFNγ levels) (Sánchez-Sánchez et al., 2025).

Unlike mice, sheep present longer gestational periods, allowing the assessment of later time points for drug administration against congenital toxoplasmosis. Furthermore, the similarities in the clinical consequences of infection during pregnancy (abortion and birth of weak offspring) between sheep and humans make sheep a suitable model for extrapolation to human disease, despite differences in placentation (Vargas-Villavicencio et al., 2016; Sánchez-Sánchez et al., 2019; Dubey et al., 2021). Although IgM is a relevant biomarker of early infection in humans, to date no studies in sheep experimental congenital models of toxoplasmosis have used IgM as a biomarker to guide therapy initiation. Studies evaluating serum IgM levels following *T. gondii* infection are very limited in sheep, with increases reported during the second week p.i (Trees et al., 1989; Lundén, 1995; Verhelst et al., 2014). In the present study, we aimed to evaluate the efficacy of BKI-1748 against congenital toxoplasmosis in sheep when the compound was administered from day 14 p.i. onwards, immediately after a significant increase in the serum-specific IgM levels. At this time point, parasite DNA had been detected in the placenta in a previous study using the same infection model (Vallejo et al., 2023). This study establishes a clinically relevant chronological framework for the administration of the therapeutic compound, translatable to human medicine, as BKI-1748 is administered once infection has been detected through serological diagnostic techniques.

## 2 Materials and methods

### 2.1 Ethics statement

Protocols were approved by the Animal Welfare Committee (Community of Madrid, Spain, PROEX 128.1/21), following Spanish and European Union legislation (Law 32/2007, R.D. 53/2013, and Council Directive 2010/63/EU).

### 2.2 Experimental design

Twenty-six pure Rasa Aragonesa breed pregnant sheep aged 18 months were selected from a commercial flock. All animals were seronegative for *T. gondii*, *N. caninum*, Border disease virus (BDV), Schmallenberg virus (SBV), *Coxiella burnetii*, and *Chlamydia abortus* as determined by ELISA. At mid-pregnancy, 90 days of gestation, 23 sheep were orally challenged with 10 *T. gondii* sporulated oocysts (stored at 4°C for 12 months after sporulation) of the *T. gondii* isolate TgShSp1 (ToxoDB genotype #3) (Sánchez-Sánchez et al., 2019) and three sheep were mock dosed with PBS (uninfected). Three out of twenty-three infected sheep (13%) experienced early abortion on days 11–12 post-infection (p.i.) and were therefore discarded from the study.

At day 14 p.i., 20 infected sheep with live fetuses were randomly distributed in two experimental groups: group 1 (G1, n = 10) that were treated orally with 10 doses at 15 mg/kg of BKI-1748 every 2 days, as in previous studies (Sánchez-Sánchez et al., 2024, 2025); and group 2 (G2, n = 10) that remained untreated. The three uninfected sheep were assigned to group 3 (G3) (**Table 1**).

**Table 1:** Experimental design.

Group	Number of pregnant sheep	Challenge (P.O.)	Treatment (P.O.)	Number of viable lambs at the beginning of treatment
G1	10^a^	10 TgShSp1 sporulated oocysts	BKI-1748^c^, 10 doses at 15 mg/kg q.o.d., starting at 14 days post-infection	23
G2	10^b^	10 TgShSp1 sporulated oocysts	None	17
G3	3	PBS	None	7

^a^ Two sheep had a total of three dead fetuses at the beginning of the treatment.

^b^ Four sheep had a total of five dead fetuses at the beginning of the treatment.

^c^ Compound was dissolved at 30 mg/mL in a vehicle containing 60% PHOSAL® 53 MCT (medium-chain triglyceride emulsion), 30% PEG400 and 10% ethanol.

P.O.: per os, orally; q.o.d.: every other day.

### 2.3 Clinical monitoring and *in vivo* collection of samples

Rectal temperatures were recorded from 0 to 14 days p.i. daily, and afterward once per week. For determination of BKI-1748 plasma concentrations, blood samples were collected from G1 into 1 mL tubes containing lithium heparin (Aquisel) following the time schedule previously described (Sánchez-Sánchez et al., 2024, 2025). Heparinized blood samples were centrifuged at 805 x *g* for 30 min at 4 °C, and plasma samples were stored at -20 °C until analysis by liquid chromatography-tandem mass spectrometry (LC-MS/MS). For evaluation of peripheral humoral immune responses in the dams, blood samples were collected on days 0, 7, 10, 12, 14, 16, 21 p.i. and then weekly until 56 days p.i. (just before delivery) in 10 mL vacutainer tubes without anticoagulant (Becton Dickinson). In lambs born alive, precolostral serum was collected immediately after delivery before ingestion of colostrum. Serum samples were stored at -80°C until analysis.

### 2.4 Viability assessment of fetuses and lambs

Transabdominal ultrasound scanning was performed weekly to evaluate fetal movements, heart beats, and the presence of hyperechoic amniotic fluid. The fetuses/lambs were classified into different categories according to the progression of gestation and their condition during the first 48 hours after birth: 1) fetuses suffering early fetal death/early abortion (i.e., until day 14 p.i.); 2) late abortions, which took place from day 15 p.i.; 3) late fetal death, in which one of the fetuses was detected as deceased by ultrasound scanning from day 15 p.i., without inducing abortion; 4) Stillborn lambs, that were found dead at the time of delivery. Deliveries at 142 days of gestation or earlier were considered premature (Wallace et al., 2021); 5) Lambs born alive but euthanized within 48 hours after birth due to the severity of clinical signs (inability or difficulty to stand, absence of voluntary suckling, and moderate hypothermia with a rectal temperature below 38 °C); 6) Viable lambs without clinical signs within 48 hours after birth. For all lambs that were born alive, the intervals (in minutes) from birth to sternal recumbency, adoption of the quadrupedal position, and first suckling were evaluated using video recordings at the animal facilities. These parameters allowed the characterization of neonatal health status and adaptive capacity after birth, facilitating the early identification of animals at higher risk of failure in passive immunity transfer and perinatal mortality (Barreto et al., 2021).

Premature and at term lambs were weighed just after birth. Birthweight of the lambs is influenced by the type of gestation (single, twin, etc.) (Barreto et al., 2021), therefore, in order to accurately compare the birthweights of the lambs, correction factors were used. These were calculated from previous experience using the birthweights of lambs born from the same breed and management; 1 for single pregnancies, 1.19 for twin pregnancies, 1.80 for triplet pregnancies and 2.11 for quadruplet pregnancies (Sánchez-Sánchez et al., 2025).

### 2.5 *Post-mortem* collection of samples

After abortion or delivery, six randomly selected cotyledons were recovered from each expelled placenta, transversally cut into 2–3 mm-thick slices, and fixed in 10% formalin for histopathological examination, whereas the remaining tissues from the cotyledons were stored at −80°C for further DNA extraction and PCR analysis. Two days after delivery, viable lambs and dams were sedated with 0.1 mg/kg of xylazine by the intravenous route (Rompun, Elanco, Monheim, Germany) and then euthanized by an intravenous overdose of embutramide and mebezonium iodide (T61, Intervet, Salamanca, Spain). Samples from brain, lungs, and semitendinosus muscle from aborted or mummified fetuses and lambs were stored at −80°C for DNA extraction and PCR analysis. In addition, a piece of brain from the offspring was fixed in 10% formalin for histopathology. Thoracic fluid was also collected from aborted fetuses and lambs born dead and maintained at −80°C for serology.

### 2.6 Study of drug pharmacokinetics in plasma and humoral immune responses against *T. gondii*

BKI-1748 plasma concentrations were determined as previously described (Hulverson et al., 2019). Calculations of maximum concentration (C_max_) for each dose, and area-under-the-curve (AUC) were determined using GraphPad Prism 8.0.1 software (San Diego, CA, USA). *Toxoplasma gondii*-specific IgG levels in dam sera were determined by a previously validated in house TgSALUVET ELISA 2.0 (cut-off for experimental conditions, RIPC ≥19.18) (López-Ureña et al., 2023). For the determination of *T. gondii*-specific IgM levels, the same methodology as for IgG was followed, using a goat/sheep anti-IgM secondary antibody (Biorad) diluted 1:5000 was used. In thoracic fluid and precolostral sera collected from aborted fetuses/dead lambs or lambs born alive, respectively, *T. gondii*-specific IgG levels were evaluated by indirect fluorescent antibody test (IFAT) (Alvarez-Garcia et al., 2003), using an anti-sheep IgG antibody (F5137, Sigma-Aldrich, Madrid, Spain) diluted 1:200 in Evans Blue. Fetal fluids and precolostral sera were diluted at 2-fold serial dilutions in PBS starting at 1:8 (for fetal fluids) and 1:50 (for precolostral sera) up to the endpoint titer. Continuous tachyzoite membrane fluorescence at a dilution of ≥1:8 for fetal fluids or ≥1:50 for precolostral sera was considered a positive reaction.

### 2.7 Parasite DNA detection/quantification and histological processing

For molecular analysis (PCR), the following samples were used: (i) six samples from different cotyledons (placental tissue) per dam; (ii) three brain tissue samples of each lamb; (iii) three samples of lung tissue per lamb; and (iv) three samples of semitendinosus muscle per lamb. Each sample consisted in 30 mg of finely cut tissue, and genomic DNA was extracted using the “Speedtools Tissue DNA Extraction Kit” (Biotools). DNA concentration for each sample was measured using a Synergy^®^ H1 multimode microplate reader (BioTek) operated with Gen5 software version 2.09.1 (BioTek). The concentration was subsequently adjusted to 100 ng/µL. Detection of *T. gondii* DNA was performed using a single-tube ITS1 nested PCR (Hurtado et al., 2001). Samples that tested positive by nested PCR were further quantified for *T. gondii* DNA through qPCR following the procedure outlined by (Castaño et al., 2016). Parasite load in tissue samples was expressed as the number of parasites per milligram of ovine tissue. In cases of congenital infection in the treated group, cotyledons and brains of the offspring were fixed for 5 days, sectioned coronally, embedded in paraffin wax, processed using standard procedures for hematoxylin and eosin (H&E) staining, and evaluated by conventional histological analysis.

### 2.8 Statistical analysis

Rectal temperatures and humoral immune responses (specific IgM and IgG) in the dams’ sera were analyzed using Two-way ANOVA of repeated measures test. The number of viable lambs, PCR-positive placentas, and PCR-positive lambs were compared across groups using Fisher’s exact test. DNA detection rates among different tissues and between viable and dead lambs were also compared using Fisher’s exact test. Lamb birth weights, IFAT titers indicating specific IgG responses in fetuses and lambs, the time intervals from birth to sternal recumbency, standing, and first suckling, as well as parasite loads in tissues, were compared using the non-parametric Kruskal–Wallis test followed by Dunn’s *post hoc* test for multiple group comparisons, and the Mann–Whitney test for pairwise analyses. AUC analysis of sheep with PCR-positive lambs and PCR-negative lambs were compared using multiple t-tests. Statistical significance for all analyses was established at P < 0.05. Differences with P values ≥ 0.05 and ≤ 0.10 were considered to have a tendency toward statistical significance. All statistical analyses were performed using GraphPad Prism 8.0.1 software (San Diego, CA, USA).

## 3 Results

### 3.1 Clinical signs and anti-*T. gondii* IgM levels prior to treatment

Rectal temperatures were significantly increased in infected sheep on days 6 (P < 0.05), 7 (P < 0.0001), 8 (P < 0.001), and 9 post-infection (p.i.) (P < 0.05) compared to uninfected sheep (**Supplementary File 1A**). Following assessment of fetal viability in the 23 infected sheep during the first 14 days p.i., three sheep (13%) that aborted on days 11–12 p.i. were excluded from the study (see Materials and Methods). Among the remaining animals, six ewes (26%) had both live and dead fetuses detected by ultrasound scanning on day 12 p.i. and were included in the evaluation of the compound efficacy, with equal distribution between the treated and untreated groups (**Table 1**). IgM levels in the serum of infected sheep began to display statistically significant differences compared to uninfected animals on day 12 p.i. (P < 0.001), with even greater significance observed by day 14 p.i. (P< 0.0001) (**Supplementary File 1B**).

### 3.2 Pharmacokinetics

BKI-1748 treatment started on day 14 p.i. The C_max_ in maternal plasma reached 4.12 ± 0.25 µM (mean ± SD) between 8 and 24 hours (mainly at 12 hours) after each BKI-1748 administration. Trough plasma concentrations were 0.7 ± 0.09 µM (mean ± SD) at 48 hours after treatment (**Supplementary File 2**). The average concentration (C_avg_) over the treatment course was 2.1 ± 1.4 µM. An AUC of 109.0 ± 7.6 h·µmol/L (mean ± SD) was observed for each BKI-1748 dose. No significant difference (P > 0.1) was observed between AUCs for sheep with PCR-positive lambs (710.7 ± 103.6 h·µmol/L) versus sheep with all PCR-negative lambs (785.0 ± 102.3 h·µmol/L). Total BKI-1748 plasma concentrations above the *in vitro* EC_50_ for *T. gondii* tachyzoites were maintained for 97% of the time during the 20 days (between days 14 to 34 p.i.). Considering that BKI-1748 shows plasma protein binding of 94.6% for sheep, the free drug concentration remains above the *in vitro* EC_50_ for 50.5% of the time during the 20-day treatment period.

### 3.3 Treatment resulted in minimal changes in specific IgM levels in challenged animals, whereas specific IgG levels were clearly reduced

**Figure 1:**
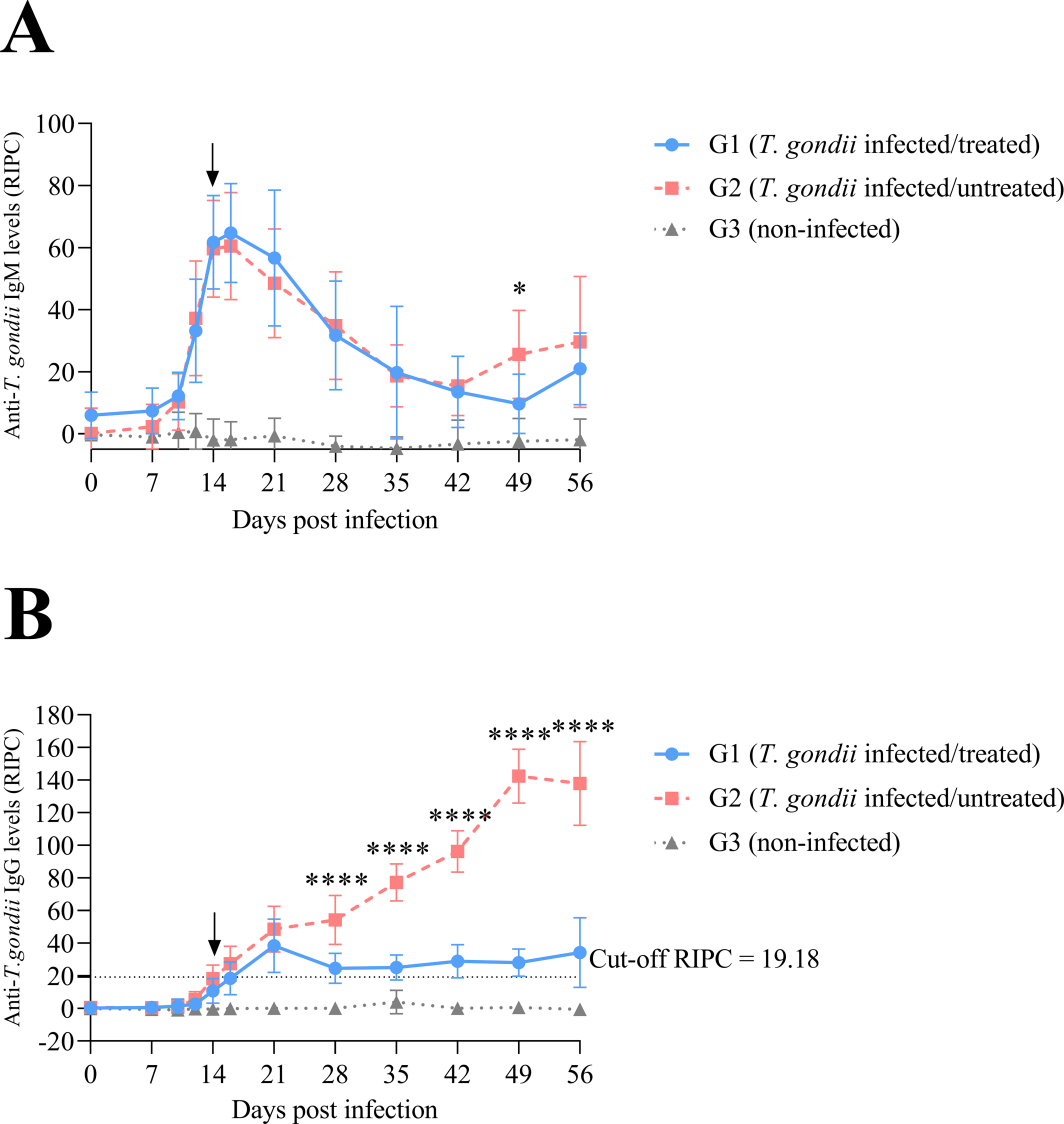
Anti-*T. gondii* IgM **(A)** and IgG **(B)** levels in dam sera. Antibody titers are expressed as relative index percent (RIPC). Each point represents the mean + S.D. at the different sampling times for each group. Black arrow points the beginning of the treatment on day 14 p.i. For significant differences between infected groups, (*) indicates P < 0.05 and (****) indicates P < 0.0001.

Regarding IgM serum levels, both *T. gondii* infected groups (treated -G1- and untreated -G2-) maintained significantly higher specific IgM levels compared to the uninfected animals, from day 16 p.i. (P < 0.0001) through day 56 p.i. (G1 vs G3, P < 0.05; G2 vs G3, P < 0.01), except on day 42 p.i. Comparing infected groups, an increase in IgM levels was observed in the untreated group (G2) on day 49 p.i. (P < 0.05) (**Figure 1A**).

Analysis of anti-*T. gondii* IgG serum levels in the dams revealed seroconversion of all infected/untreated sheep (G2) by day 21 p.i. with a progressive increase of IgG levels that continued through the end of the study (day 56 p.i.). In infected/treated sheep (G1), 8 out of 10 animals had seroconverted by day 21 p.i., with another sheep seroconverting by day 35 p.i., while the remaining animal (sheep 12) did not seroconvert throughout the study. The IgG levels in infected/treated sheep (G1) remained slightly above the cut-off value during the follow-up period.

In G1 (infected/treated sheep), no significant differences in specific IgG levels were observed between sheep that transmitted the infection congenitally and those that did not. When both infected groups were compared, sheep in G1 (infected/treated) exhibited significantly lower IgG levels than those in G2 (infected/untreated) from day 28 p.i. through the end of the experiment on day 56 p.i. (P < 0.0001) (**Figure 1B**).

### 3.4 Treatment in the challenge group led to an increased number of viable lambs, which exhibited greater neonatal vitality but low birth weights

**Figure 2:**
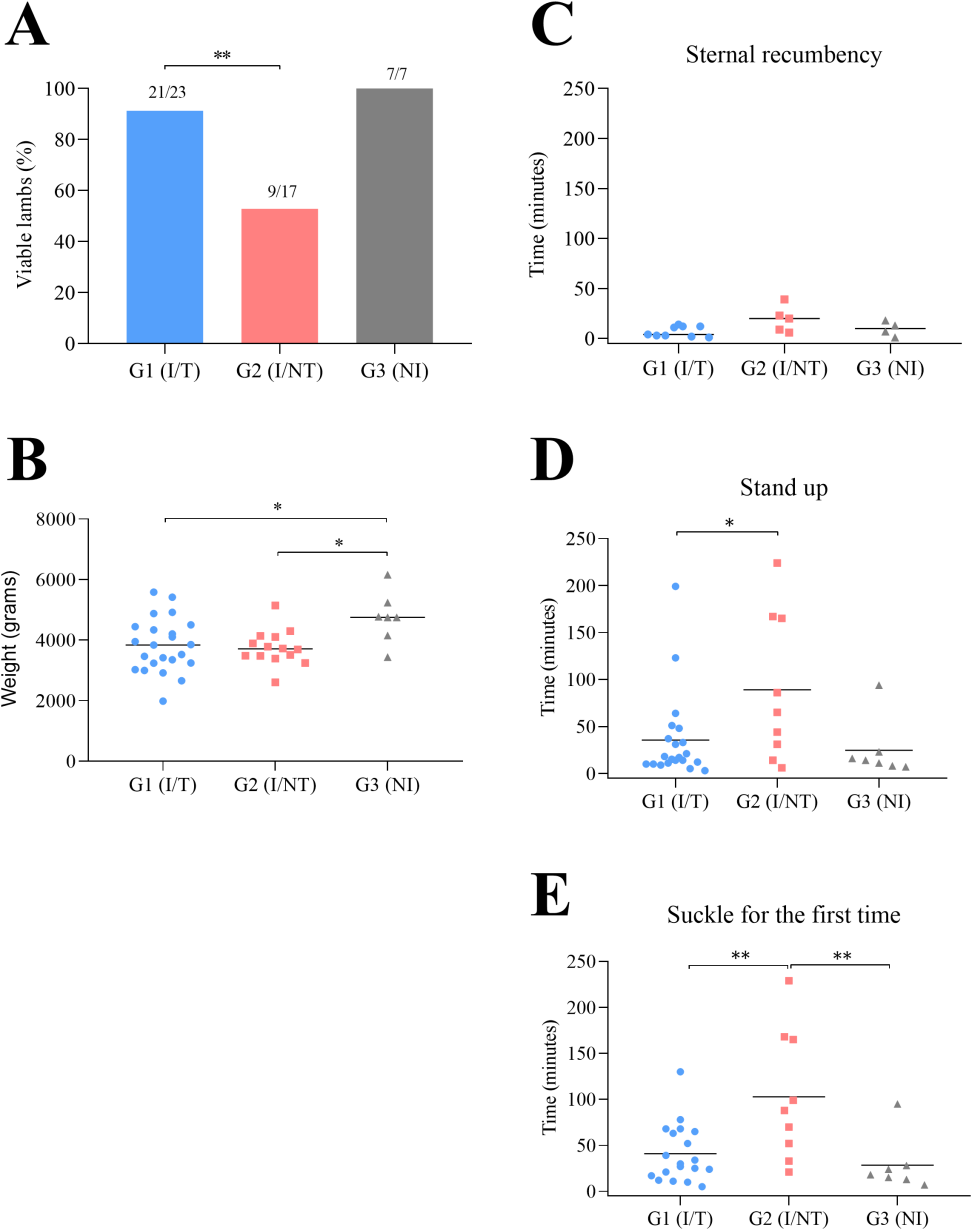
Proportions of viable lambs **(A)**, birthweight of the lambs **(B)** and viability of lambs at birth **(C–E)** from *T. gondii* infected (treated or not) and uninfected sheep. In **(B)**, the birthweights of lambs (including stillbirths, lambs euthanized during 48 hours after birth and viable lambs) are shown. Birthweight was corrected applying a correction factor depending on the number of lambs per sheep (see Material and Methods section). In **(C, D, E)**, times (in minutes) from birth to achieve sternal recumbency **(A)**, to stand up **(B)**, and to suckle for the first time **(C)** are shown. For significant differences, (*) indicates P < 0.05 and (**) indicates P < 0.01.

Data on pregnancy outcomes in the three experimental groups are presented in **Table 2** and detailed in **Supplementary File 3**. In the infected, untreated group (G2), only 52.9% (9/17) of the lambs were born viable. By contrast, in the infected and treated group (G1), no abortions, intrauterine fetal deaths, or stillbirths were recorded, and of the 23 lambs born, 21 (91.3%) were classified as viable. As expected, all three sheep in the uninfected group (G3) gave birth to viable lambs (7/7; 100 %) (**Table 2**). Thus, a significantly higher percentage of viable lambs were found in G1 (infected/treated) compared to G2 (infected/untreated) (P < 0.01) (**Figure 2A**).

**Table 2:** Reproductive outcomes and parasite DNA identification in placental and offspring target tissues.

Group		Group 1 (infected/treated)				Group 2 (infected/untreated)				Group 3 (uninfected)			
Animals		Dams		Fetuses/lambs		Dams		Fetuses/lambs		Dams		Fetuses/lambs	
Pregnancy outcomes^a^		n^b^	PCR^c^	n^b^	PCR^c^	n^b^	PCR^c^	n^b^	PCR^c^	n^b^	PCR^c^	n^b^	PCR^c^
Late abortion		–	–	–	–	1/10 (10%)	0/10 (0%)	1/17 (5.9%)	1/16 (6.2%)	–	–	–	–
Delivery	Late death	10/10 (100%)	2/10 (20%)	–	–	9/10 (90%)^e^	7/8 (87.5%)^f^	2/17 (11.8%)^d^	1/16 (6.2%)	3/3 (100%)	0/3 (0%)	–	–
	Stillbirths			–	–			1/17 (5.9%)	1/16 (6.2%)			–	–
	Euthanized during 48 hours after birth			2/23 (8.7%)	1/23 (4.3%)			4/17 (23.5%)	4/16 (25.1%)			–	–
	Viable			21/23 (91.3%)	3/23 (13%)			9/17 (52.9%)	9/16 (56.3%)			7/7 (100%)	0/7 (0%)

^a^ The pregnancy outcomes were categorized as follows: Late abortions, which took place from day 15 p.i. onwards (47 days p.i. in sheep suffering late abortion in group 2); Late fetal death, in which one of the fetuses was detected as deceased by ultrasound scanning from day 15 p.i. onwards, without inducing abortion (days 34 and 48 p.i. in the fetuses suffering late death in group 2); Stillborn lambs, that were found dead at the time of delivery; Lambs that were born alive but were euthanized within 48 hours after birth due to severity of the clinical signs; Viable lambs, without clinical signs within 48 hours after birth.

^b^ Number of dams/total number of dams (percentage in brackets); Number of fetuses or lambs/total number of fetuses or lambs (percentage in brackets).

^c^ Parasite DNA detection by nested PCR in placental tissues (cotyledons) from dams and in tissues from fetuses/lambs. Number of PCR-positive placentas/total number of placentas analyzed (percentage in brackets); number of PCR-positive fetuses or lambs/total number of fetuses or lambs analyzed (percentage in brackets). A placenta is considered positive when one or more of the six cotyledons analyzed are PCR-positive. A fetus/lamb is considered positive when one or more replicates from any of the three tissues analyzed (brain, lung, and muscle) are PCR-positive. For detailed data, see **Supplementary File 3**.

^d^ Tissues from one fetus that suffered late death were too autolytic, resulting in degraded DNA.

^e^ Of the nine sheep carrying viable fetuses, four experienced premature delivery (≤142 days of gestation).

^f^ The cotyledons from one sheep were not available for parasite DNA detection due to placentophagy.

(–) indicates the absence of dams or fetuses/lambs in the corresponding category for pregnancy outcome.

Regarding the weight of lambs upon birth (including stillborn, lambs euthanized during 48 hours after birth, and viable lambs), those from the infected/treated group (G1) and infected/untreated group (G2) exhibited significantly lower birthweights (20.8% reduction in lambs from both infected groups) compared to G3 (uninfected sheep) (P < 0.05) (**Figure 2B**).

With respect to viability of live-born lambs, comparing both infected groups, lambs in G1 (infected/treated) stood up (P < 0.05) and attempted to suckle (P < 0.01) sooner than those in G2 (infected/untreated), with a tendency toward significance in the time to achieve sternal recumbency (P = 0.055) (**Figures 2C–E**). Compared with lambs from the uninfected group (G3), lambs in G1 (infected/treated) showed no significant differences in the times to achieve sternal recumbency, stand, or suckle overall; however, the time to stand up tended to be longer (P = 0.08) and the time to suckle was significantly longer (P < 0.01) in G2 (infected/untreated) lambs. Thus, lambs in the infected/treated group exhibited improved early neonatal vitality than infected/untreated lambs.

### 3.5 Treatment reduced congenital infection rates, and congenitally infected lambs exhibited reduced birth weights

In the infected and untreated group (G2), all lambs that died before or immediately after birth, as well as those born viable, tested PCR-positive (16/16) (**Table 2**). The detection rates and parasite loads in the lung were significantly higher than those in the brain and semitendinosus muscle (P < 0.0001), whereas no differences were observed between the brain and semitendinosus muscle (**Supplementary File 4**). Seropositive IgG titers were detected in all dead fetuses (2/2) and in 87.5% (12/14) lambs, including those that died before or immediately after birth and those born viable (**Supplementary File 3**). No differences in parasite DNA detection rates, parasite loads, or IFAT titers were observed between stillborn and viable lambs. In the infected and treated group (G1), 4 out of 23 lambs (17.4%) tested PCR-positive; two of these PCR-positive lambs exhibited glial foci in the midbrain (**Table 2**; **Supplementary File 3**). PCR-positive lambs were born to 3 of the 10 sheep (30%; sheep 1, 5, and 9) in this group. In the PCR-positive lambs, the detection rates in the lungs and brain were significantly higher than those in the semitendinosus muscle (P < 0.0001). Seropositive IgG titers were detected in 13% (3/23) of the lambs (**Supplementary File 3**).

**Figure 3:**
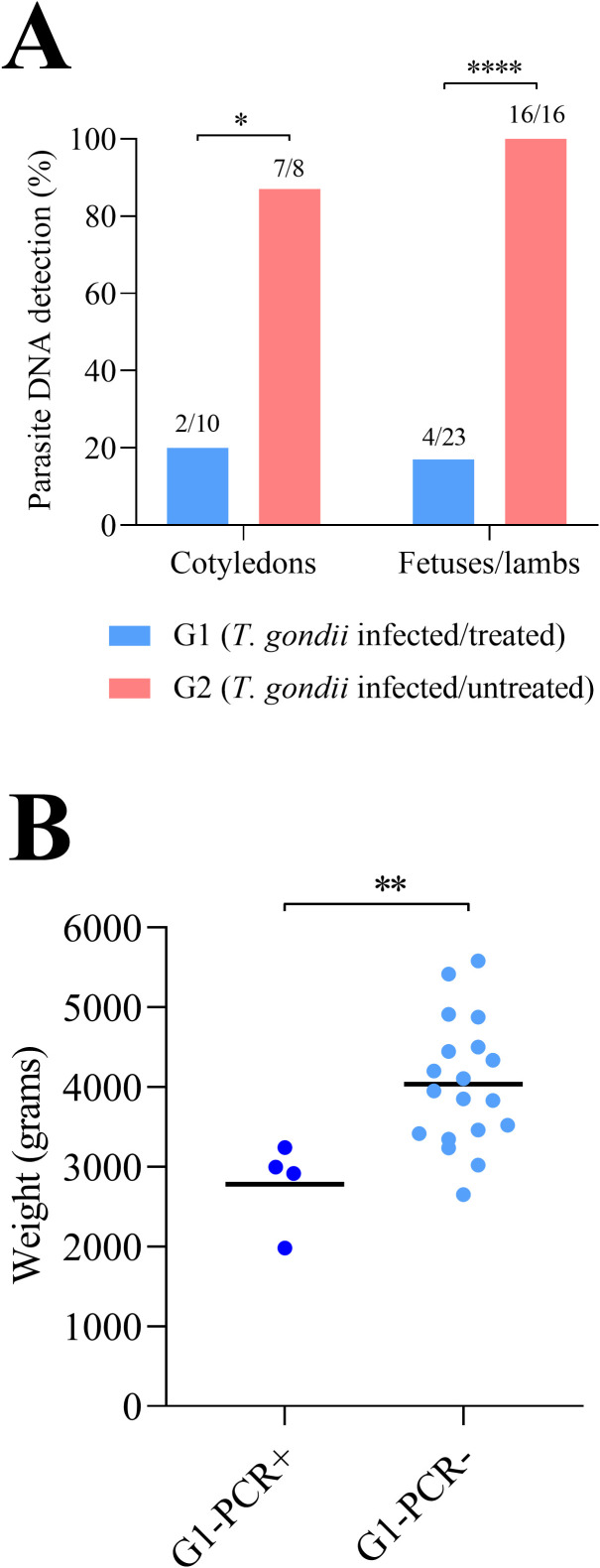
Proportions of PCR-positive placentas and fetuses/lambs **(A)** and birth weight of lambs from infected and treated sheep **(B)**. For significant differences, (*) indicates P < 0.05, (**) indicates P < 0.01 and (****) indicates P < 0.0001.

Comparing infected groups, lower proportion of PCR-positive lambs (100% in G2 *vs* 17.4% in G1) (**Figure 3A**), IFAT titers (**Supplementary File 3**), and parasite loads (**Supplementary File 5**) were found in G1 compared to G2 (P < 0.0001). For PCR-positive lambs in G1, higher detection rates were observed in the brain (P < 0.05) and lower detection rates in the semitendinosus muscle (P < 0.01), compared to lambs in G2. Lambs in group G1 (infected/treated) that tested positive by PCR exhibited a significantly lower birth weight (30.1% reduction) compared to PCR-negative lambs within the same group (P < 0.01) (**Figure 3B**). Conversely, no significant difference in birth weight was detected between PCR-negative lambs in G1 and those born to uninfected sheep (G3).

In the cotyledons (analogous to placentas), the parasite DNA detection (**Figure 3A**) and the parasite loads (**Supplementary File 5**) were significantly lower in treated (G1) than in untreated sheep (G2). The two sheep with PCR-positive placentas in G1 also had PCR-positive fetuses, and one of them showed necrotic foci in the cotyledons (**Supplementary File 3**). Examination of placental and fetal samples from the uninfected group (G3) confirmed the absence of *T. gondii*–specific DNA.

## 4 Discussion

The controversial efficacy of folate inhibitors in humans, and the fact that up to 37% of treated patients require treatment discontinuation or therapy modification due to adverse effects, renders these compounds suboptimal for the treatment of toxoplasmosis during pregnancy (Robert-Gangneux, 2014; Ben-Harari et al., 2017; Shammaa et al., 2021). Therefore, there is an urgent need for development of new well-developed therapeutic compounds against congenital toxoplasmosis. The effectiveness of prenatal interventions in mitigating the clinical outcomes of congenital infection depends on how promptly treatment is initiated following maternal *T. gondii* infection (Thiébaut et al., 2007; Mandelbrot et al., 2018). However, accurately determining the timing of infection is challenging in clinical practice. In this context, serum IgM seroconversion is a widely used serological tool in monitoring programs as a serological marker for the diagnosis of recent infection in pregnant women (Konstantinovic et al., 2019; Fricker-Hidalgo et al., 2020). The efficacy of the compound BKI-1748, which exhibits a favorable *in vivo* safety profile and potential acceptable cardiovascular safety in humans, has recently been evaluated in pregnant sheep models of toxoplasmosis when administered at early time points demonstrating high effectiveness in reducing lamb mortality and preventing vertical transmission (Sánchez-Sánchez et al., 2024, 2025). In this study, BKI-1748 treatment was initiated on day 14 post-infection (p.i.), when sheep exhibited a significant increase in the serum-specific IgM levels and parasite DNA was detectable in the placenta, as demonstrated in a previous study using the same ovine infection model employed here (Vallejo et al., 2023).

Regarding the features of the infection model prior to drug administration, the sheep had already experienced the peak of fever, which occurred between days 6 and 9 p.i., and 13% of the animals had presented early abortion on days 11–12 p.i. The early aborted fetuses were PCR-negative, as expected in this infection model (Sánchez-Sánchez et al., 2019; Vallejo et al., 2023; Sánchez-Sánchez et al., 2025; Regidor-Cerrillo et al., 2026). By day 12 p.i., 8 of the 54 fetuses from infected sheep (14.8%) had died and remained *in utero* as mummified fetuses until delivery in multiple pregnancies with surviving co-fetuses. These findings are consistent with previous reports using both experimental low-dose oocyst infections (Castaño et al., 2016; Chiebao et al., 2019; Sánchez-Sánchez et al., 2025) and in natural infections (Giadinis et al., 2011; Edwards and Dubey, 2013; Lefebvre, 2015; Dorsch et al., 2022; Withoeft et al., 2024).

Treatment with BKI-1748 did not alter serum IgM levels in the dams, in agreement with previous findings in *T. gondii*–infected mice treated with commonly used drugs (Alvarado-Esquivel et al., 2011). In the untreated sheep, the increase in specific IgM levels around the time of delivery (49 days p.i.; 139 days of gestation) may be associated with reactivation of the chronic infection driven by peripartum immunosuppression (Vargas-Villavicencio et al., 2022). Regarding IgG levels in the dams, infected but untreated sheep seroconverted at 21 days p.i., as previously described (López-Ureña et al., 2023; Sánchez-Sánchez et al., 2025). In the treated sheep, although 80% of them were seropositive on day 21 p.i., much lower IgG levels were observed from day 28 p.i. onward compared to the untreated sheep, likely reflecting reduced parasite proliferation. In a previous study where BKI-1748 treatment was initiated at day 7 p.i., reduced IgG levels were already evident at day 21 p.i (Sánchez-Sánchez et al., 2025); however, in the present study (using the same methodology), this reduction in IgG levels was only observed from day 28 p.i. due to the delayed onset of treatment.

Abortions or fetal death during late pregnancy, stillborn lambs, and lambs showing early postnatal clinical signs had been observed earlier in 0-13%, 10-18%, and 10-18% of offspring, respectively, from sheep infected at mid-pregnancy with 10 sporulated oocysts of the TgShSp1 isolate (Sánchez-Sánchez et al., 2019; Vallejo et al., 2023; Sánchez-Sánchez et al., 2025; Regidor-Cerrillo et al., 2026). Similar offspring survival outcomes were observed in the infected, untreated sheep in the present study, as well as in naturally infected animals (Dubey et al., 2020). Additionally, congenital *T. gondii* infection following challenge with a low dose of oocysts resulted in reduced birthweight of the lambs, as previously reported (Buxton et al., 1996; Sánchez-Sánchez et al., 2025), as well as decreased neonatal vitality (manifested as longer times required to stand up and to suckle). Both clinical features have also been described in humans (Bollani et al., 2022); however, some long-term clinical manifestations described in humans could not be evaluated in this study. The occurrence of stillborn and lambs exhibiting early postnatal clinical signs, together with the reduced birthweight and decreased neonatal vitality, may be attributable to tachyzoite replication of the TgShSp1 isolate, which exhibits a high tropism for placental and fetal tissues (Vallejo et al., 2023). In sheep infected and treated with BKI-1748, 91.3% of lambs were born viable, compared to 52.9% in infected, untreated sheep. Lambs born to BKI-1748 treated sheep exhibited normal vitality at birth, with times to achieve sternal recumbency, stand up, and suckle for the first time as in lambs born to uninfected sheep, suggesting that the pharmacological intervention, even when administered late, significantly enhances early postnatal vitality in lambs.

Based on parasite DNA detection rates and antibody titers in lambs, the sheep-to-lamb transmission rate was 100% in the untreated/infected group with 10 TgShSp1 oocysts at 90 days of gestation, as previously described (Sánchez-Sánchez et al., 2019, 2025). Administration of BKI-1748 from day 7 p.i. provided complete protection against congenital transmission (Sánchez-Sánchez et al., 2025); however, when administration was started on day 14 p.i., 17.4% of the lambs (one lamb euthanized during 48 hours after birth and three viable lambs) were born congenitally infected (PCR-positive). An additional lamb (4.3%) in the treated/infected group was seropositive by IFAT but PCR-negative which may indicate that, although it was exposed to the parasite, it was able to control the infection. PCR-positive lambs born to infected and treated sheep had lower birthweights than PCR-negative lambs within the same group; however, this finding should be interpreted with caution given the small number of congenitally infected animals. This effect may be attributable to tissue damage resulting from parasite replication in fetal tissues, as glial foci were observed in two of the four PCR-positive lambs in this group. Although the subgroup of treated sheep that transmitted the infection was limited in size, no significant differences were observed in IgG or BKI-1748 plasma levels between sheep that transmitted the infection and those that did not, suggesting that congenital transmission may be largely determined by the placental passage of BKI-1748. In this regard, a previous study that evaluated BKI-1748 levels in fetal plasma following a single dose reported a fetus-to-dam BKI-1748 ratio of 0.27 (Sánchez-Sánchez et al., 2024). It also showed that, considering a plasma protein binding of 94.6% for BKI-1748, the unbound (active) drug concentration at fetal C_max_ (total, 0.6 µM; unbound, 0.032 µM) falls below the EC_50_ for *T. gondii* (0.063 µM) (Hulverson et al., 2019; Imhof et al., 2021). In the present study, BKI-1748 plasma levels in the dams were similar to those reported in previous studies using the same dosing regimen (Sánchez-Sánchez et al., 2024, 2025). In our study, an extended treatment regimen with BKI-1748 (10 doses of 15 mg/kg administered every two days) was evaluated because BKIs exhibit an *in vitro* parasitostatic effect (Müller et al., 2017) and previous studies in sheep, in which BKI-1748 was administered at 2 and 7 days post-infection, demonstrated highly satisfactory protection against congenital toxoplasmosis (Sánchez-Sánchez et al., 2024, 2025). However, in the present study, treatment was initiated after the parasite had already colonized the placenta and fetal exposure to free (unbound) drug may have been suboptimal. These factors likely contributed to the failure to prevent congenital transmission in several lambs and to the reduced birth weight observed in congenitally infected lambs.

In conclusion, drug trials in animal models of congenital toxoplasmosis in which therapy is initiated at the time of peak IgM levels and after placental colonization has occurred are highly relevant for anticipating the potential usefulness of drugs in human medicine. BKI-1748 treatment in pregnant sheep infected at mid-pregnancy, with administration started on day 14 p.i. onwards and maintained for 20 days, significantly improved lamb viability (91% viable lambs vs. 52% in the untreated group) and reduced congenital infection (17% congenitally infected lambs vs. 100% in the untreated group). Future studies should explore: i) interspecies differences between sheep and humans in placental drug transfer and fetal pharmacokinetics of BKI-1748, ii) novel dosing regimens of BKI-1748 in animals models aimed at maximizing fetal drug exposure, as well as the evaluation of BKIs with improved placental transfer, while prioritizing safety in all approaches (Hulverson et al., 2019; Choi et al., 2020), iii) efficacy of BKIs administered once the parasite has been detected in amniotic fluid (Marques et al., 2012), and iv) efficacy of BKIs against non-canonical *T. gondii* isolates, which predominate in South America and are associated with severe clinical outcomes in humans (de Lima Bessa et al., 2021). To date, efficacy studies of BKIs in animal models of congenital toxoplasmosis have been conducted exclusively using the Type II *T. gondii* lineage, which predominates in Europe and North America.

## Data Availability

The original contributions presented in the study are included in the article/**Supplementary Material**. Further inquiries can be directed to the corresponding authors.

## Supplementary material

Supplementary File 1 Rectal temperatures **(A)** and serum anti-*T. gondii*-IgM levels **(B)** during the first two weeks post-infection.

Supplementary File 2 Plasma levels of BKI-1748 in infected and treated dams.

Supplementary File 3 Individual data of clinical outcome and infection status of the offspring.

Supplementary File 4 Parasite loads in tissues from fetuses/lambs in the infected/untreated group (G2).

Supplementary File 5 Box plots illustrating parasite burdens in fetal/lamb tissues and placental cotyledons.

## References

[B1] Aguirre A. A. Longcore T. Barbieri M. Dabritz H. Hill D. Klein P. N. . (2019). The one health approach to toxoplasmosis: Epidemiology, control, and prevention strategies. Ecohealth 16, 378–390. doi: 10.1007/s10393-019-01405-7. PMID: 30945159 PMC6682582

[B2] Alday P. H. Doggett J. S. (2017). Drugs in development for toxoplasmosis: Advances, challenges, and current status. Drug Des. Devel. Ther. 11, 273–293. doi: 10.2147/DDDT.S60973. PMID: 28182168 PMC5279849

[B3] Alvarado-Esquivel C. Niewiadomski A. Schweickert B. Liesenfeld O. (2011). Antiparasitic treatment suppresses production and avidity of Toxoplasma gondii-specific antibodies in a murine model of acute infection. Eur. J. Microbiol. Immunol. 1, 249–255. doi: 10.1556/EuJMI.1.2011.3.9. PMID: 24516731 PMC3906621

[B4] Alvarez-Garcia G. Collantes-Fernandez E. Costas E. Rebordosa X. Ortega-Mora L. M. (2003). Influence of age and purpose for testing on the cut-off selection of serological methods in bovine neosporosis. Vet. Res. 34, 341–352. doi: 10.1051/vetres:2003009. PMID: 12791243

[B5] Anghel N. Winzer P. A. Imhof D. Müller J. Langa X. Rieder J. . (2020). Comparative assessment of the effects of bumped kinase inhibitors on early zebrafish embryo development and pregnancy in mice. Int. J. Antimicrob. Agents 56, 106099. doi: 10.1016/j.ijantimicag.2020.106099. PMID: 32707170 PMC8582287

[B6] Barreto J. V. P. Pertile S. F. N. de Almeida Rego F. C. Patelli T. H. C. Nascimento S. T. Lorenzetti E. . (2021). Prediction of vitality and survival of newborn lambs using a modified Apgar score. Appl. Anim. Behav. Sci. 238, 105281. doi: 10.1016/j.applanim.2021.105281. PMID: 38826717

[B7] Ben-Harari R. R. Goodwin E. Casoy J. (2017). Adverse event profile of pyrimethamine-based therapy in toxoplasmosis: A systematic review. Drugs R. 17, 523–544. doi: 10.1007/s40268-017-0206-8. PMID: 28879584 PMC5694419

[B8] Binquet C. Lejeune C. Seror V. Peyron F. Bertaux A. Scemama O. . (2019). The cost-effectiveness of neonatal versus prenatal screening for congenital toxoplasmosis. PloS One 14, e0221709. doi: 10.1371/journal.pone.0221709. PMID: 31532766 PMC6750576

[B9] Bollani L. Auriti C. Achille C. Garofoli F. De Rose D. U. Meroni V. . (2022). Congenital toxoplasmosis: The state of the art. Front. Pediatr. 10. doi: 10.3389/fped.2022.894573. PMID: 35874584 PMC9301253

[B10] Buxton D. Brebner J. Wright S. Maley S. W. Thomson K. M. Millard K. (1996). Decoquinate and the control of experimental ovine toxoplasmosis. Vet. Rec. 138, 434–436. doi: 10.1136/vr.138.18.434. PMID: 8735260

[B11] Buxton D. Thomson K. Maley S. Wright S. Bos H. J. (1991). Vaccination of sheep with a live incomplete strain (s48) of Toxoplasma gondii and their immunity to challenge when pregnant. Vet. Rec. 129, 89–93. doi: 10.1136/vr.129.5.89. PMID: 1926725

[B12] Cardew E. M. Verlinde C. L. Pohl E. (2018). The calcium-dependent protein kinase 1 from Toxoplasma gondii as target for structure-based drug design. Parasitology 145, 210–218. doi: 10.1017/S0031182017001901. PMID: 29191253 PMC5964469

[B13] Castaño P. Fuertes M. Regidor-Cerrillo J. Ferre I. Fernandez M. Ferreras M. C. . (2016). Experimental ovine toxoplasmosis: Influence of the gestational stage on the clinical course, lesion development and parasite distribution. Vet. Res. 47, 43. doi: 10.1186/s13567-016-0327-z. PMID: 26983883 PMC4793618

[B14] Chiebao D. P. Pena H. F. Passarelli D. Santín T. Pulz L. H. Strefezzi R. F. . (2019). Congenital transmission of Toxoplasma gondii after experimental reinfection with Brazilian typical strains in chronically infected sheep. Front. Vet. Sci. 6. doi: 10.3389/fvets.2019.00093. PMID: 31001545 PMC6454189

[B15] Choi R. Hulverson M. A. Huang W. Vidadala R. S. Whitman G. R. Barrett L. K. . (2020). Bumped kinase inhibitors as therapy for apicomplexan parasitic diseases: Lessons learned. Int. J. Parasitol. 50, 413–422. doi: 10.1016/j.ijpara.2020.01.006. PMID: 32224121 PMC8565993

[B16] de Lima Bessa G. de Almeida Vitor R. W. dos Santos Martins-Duarte E. (2021). Toxoplasma gondii in South America: A differentiated pattern of spread, population structure and clinical manifestations. Parasitol. Res. 120, 3065–3076. doi: 10.1007/s00436-021-07282-w. PMID: 34390383

[B17] Dorsch M. A. Francia M. E. Tana L. R. González F. C. Cabrera A. Calleros L. . (2022). Diagnostic investigation of 100 cases of abortion in sheep in Uruguay: 2015-2021. Front. Vet. Sci. 9. doi: 10.3389/fvets.2022.904786. PMID: 35664842 PMC9161216

[B18] Dubey J. P. (2021). Toxoplasmosis of Animals and Humans (Florida: CRC Press).

[B19] Dubey J. P. Murata F. H. A. Cerqueira-Cezar C. K. Kwok O. C. H. Su C. (2020). Economic and public health importance of Toxoplasma gondii infections in sheep: 2009-2020. Vet. Parasitol. 286, 109195. doi: 10.1016/j.vetpar.2020.109195. PMID: 32979682

[B20] Dubey J. P. Murata F. Cerqueira-Cézar C. K. Kwok O. Villena I. (2021). Congenital toxoplasmosis in humans: An update of worldwide rate of congenital infections. Parasitology 148, 1406–1416. doi: 10.1017/S0031182021001013. PMID: 34254575 PMC11010219

[B21] Dunay I. R. Gajurel K. Dhakal R. Liesenfeld O. Montoya J. G. (2018). Treatment of toxoplasmosis: Historical perspective, animal models, and current clinical practice. Clin. Microbiol. Rev. 31, 00057–00017. doi: 10.1128/CMR.00057-17. PMID: 30209035 PMC6148195

[B22] Edwards J. F. Dubey J. P. (2013). Toxoplasma gondii abortion storm in sheep on a Texas farm and isolation of mouse virulent atypical genotype T. gondii from an aborted lamb from a chronically infected ewe. Vet. Parasitol. 192, 129–136. doi: 10.1016/j.vetpar.2012.09.037. PMID: 23099088

[B23] Elsheikha H. M. Marra C. M. Zhu X. (2020). Epidemiology, pathophysiology, diagnosis, and management of cerebral toxoplasmosis. Clin. Microbiol. Rev. 34, 00115–00119. doi: 10.1128/CMR.00115-19. PMID: 33239310 PMC7690944

[B24] Fricker-Hidalgo H. Bailly S. Brenier-Pinchart M. Dard C. Jean D. Coston A. . (2020). How to estimate time of infection with Toxoplasma gondii in pregnant women. Use of specific IgG and IgM kinetics by 7 techniques on 691 sera. Diagn. Microbiol. Infect. Dis. 96, 114987. doi: 10.1016/j.diagmicrobio.2020.114987. PMID: 32005405

[B25] Garweg J. G. Kieffer F. Mandelbrot L. Peyron F. Wallon M. (2022). Long-term outcomes in children with congenital toxoplasmosis—a systematic review. Pathogens 11, 1187. doi: 10.3390/pathogens11101187. PMID: 36297244 PMC9610672

[B26] Giadinis N. D. Terpsidis K. Diakou A. Siarkou V. Loukopoulos P. Osman R. . (2011). Massive toxoplasma abortions in a dairy sheep flock and therapeutic approach with different doses of sulfadimidine. Turk. J. Vet. Anim. Sci. 35, 207–211. doi: 10.3906/vet-0910-170

[B27] Gilbert R. E. Peckham C. S. (2002). Congenital toxoplasmosis in the United Kingdom: To screen or not to screen? J. Med. Screen 9, 135–141. doi: 10.1136/jms.9.3.135. PMID: 12370327

[B28] Gutiérrez-Expósito D. Tejerina F. Gutiérrez J. Fernández-Escobar M. Ortega-Mora L. M. Mantecón A. R. . (2021). Direct economic losses of Toxoplasma gondii abortion outbreaks in two Spanish sheep flocks. Vet. Parasitol. Reg. Stud. Rep. 26, 100623. doi: 10.1016/j.vprsr.2021.100623. PMID: 34879935

[B29] Hulverson M. A. Bruzual I. McConnell E. V. Huang W. Vidadala R. S. Choi R. . (2019). Pharmacokinetics and *in vivo* efficacy of pyrazolopyrimidine, pyrrolopyrimidine, and 5-aminopyrazole-4-carboxamide bumped kinase inhibitors against toxoplasmosis. J. Infect. Dis. 219, 1464–1473. doi: 10.1093/infdis/jiy664. PMID: 30423128 PMC6467197

[B30] Hurtado A. Aduriz G. Moreno B. Barandika J. GarcÍa-Pérez A. L. (2001). Single tube nested PCR for the detection of Toxoplasma gondii in foetal tissues from naturally aborted ewes. Vet. Parasitol. 102, 17–27. doi: 10.1016/s0304-4017(01)00526-x. PMID: 11705648

[B31] Imhof D. Anghel N. Winzer P. Balmer V. Ramseier J. Hänggeli K. . (2021). *In vitro* activity, safety and *in vivo* efficacy of the novel bumped kinase inhibitor BKI-1748 in non-pregnant and pregnant mice experimentally infected with Neospora caninum tachyzoites and Toxoplasma gondii oocysts. Int. J. Parasitol. Drugs Drug Resist. 16, 90–101. doi: 10.1016/j.ijpddr.2021.05.001. PMID: 34030110 PMC8144743

[B32] Kieschnick H. Wakefield T. Narducci C. A. Beckers C. (2001). Toxoplasma gondii attachment to host cells is regulated by a calmodulin-like domain protein kinase. J. Biol. Chem. 276, 12369–12377. doi: 10.1074/jbc.M011045200. PMID: 11154702

[B33] Konstantinovic N. Guegan H. Stäjner T. Belaz S. Robert-Gangneux F. (2019). Treatment of toxoplasmosis: Current options and future perspectives. Food Waterborne Parasitol. 15, e00036. doi: 10.1016/j.fawpar.2019.e00036. PMID: 32095610 PMC7033996

[B34] Lefebvre R. C. (2015). Foetal mummification in the major domestic species: Current perspectives on causes and management. Vet. Med. 6, 233–244. doi: 10.2147/VMRR.S59520. PMID: 30101110 PMC6067784

[B35] López-Ureña N. M. Calero-Bernal R. Vázquez-Calvo Á. Sánchez-Sánchez R. Ortega-Mora L. M. Álvarez-García G. (2023). A comparative study of serological tests used in the diagnosis of Toxoplasma gondii infection in small ruminants evidenced the importance of cross-reactions for harmonizing diagnostic performance. Res. Vet. Sci. 165, 105052. doi: 10.1016/j.rvsc.2023.105052. PMID: 37864907

[B36] Lourido S. Shuman J. Zhang C. Shokat K. M. Hui R. Sibley L. D. (2010). Calcium-dependent protein kinase 1 is an essential regulator of exocytosis in Toxoplasma. Nature 465, 359–362. doi: 10.1038/nature09022. PMID: 20485436 PMC2874977

[B37] Lourido S. Tang K. Sibley L. D. (2012). Distinct signalling pathways control Toxoplasma egress and host-cell invasion. EMBO J. 31, 4524–4534. doi: 10.1038/emboj.2012.299. PMID: 23149386 PMC3545288

[B38] Lundén A. (1995). Immune responses in sheep after immunization with Toxoplasma gondii antigens incorporated into iscoms. Vet. Parasitol. 56, 23–35. doi: 10.1016/0304-4017(94)00670-8. PMID: 7732647

[B39] Maldonado Y. A. Read J. S. (2017). Diagnosis, treatment, and prevention of congenital toxoplasmosis in the United States. Pediatrics 139, e20163860. doi: 10.1542/peds.2016-3860. PMID: 28138010

[B40] Mandelbrot L. (2020). Congenital toxoplasmosis: What is the evidence for chemoprophylaxis to prevent foetal infection? Prenat. Diagn. 40, 1693–1702. doi: 10.1002/pd.5758. PMID: 32453454

[B41] Mandelbrot L. Kieffer F. Sitta R. Laurichesse-Delmas H. Winer N. Mesnard L. . (2018). Prenatal therapy with pyrimethamine sulfadiazine vs spiramycin to reduce placental transmission of toxoplasmosis: A multicenter, randomized trial. Am. J. Obstet. Gynecol 219, 386.e1–386.e9. doi: 10.1016/j.ajog.2018.05.031. PMID: 29870736

[B42] Marques P. X. O’Donovan J. Williams E. J. Gutierrez J. Worrall S. McElroy M. . (2012). Detection of Toxoplasma gondii antigens reactive with antibodies from serum, amniotic, and allantoic fluids from experimentally infected pregnant ewes. Vet. Parasitol. 185, 91–100. doi: 10.1016/j.vetpar.2011.10.028. PMID: 22088616

[B43] Montgomery J. A. Alday P. H. Choi R. Khim M. Staker B. L. Hulverson M. A. . (2025). Bumped kinase inhibitors inhibit both Toxoplasma gondii MAPKL1 and CDPK1. ACS Infect. Dis. 11, 1552–1562. doi: 10.1021/acsinfecdis.5c00051. PMID: 40407244 PMC12172689

[B44] Montoya J. G. (2018). Systematic screening and treatment of toxoplasmosis during pregnancy: Is the glass half full or half empty? Am. J. Obstet. Gynecol 219, 315–319. doi: 10.1016/j.ajog.2018.08.001. PMID: 30269768

[B45] Montoya J. G. Rosso F. (2005). Diagnosis and management of toxoplasmosis. Clin. Perinatol. 32, 705–726. doi: 10.1016/j.clp.2005.04.011. PMID: 16085028

[B46] Müller J. Aguado-Martínez A. Balmer V. Maly D. J. Fan E. Ortega-Mora L. . (2017). Two novel calcium-dependent protein kinase 1 inhibitors interfere with vertical transmission in mice infected with Neospora caninum tachyzoites. Antimicrob. Agents Chemother. 61, 2324. doi: 10.1128/AAC.02324-16. PMID: 28137808 PMC5365725

[B47] Müller J. Anghel N. Imhof D. Hänggeli K. Uldry A. Braga-Lagache S. . (2022). Common molecular targets of a quinolone based bumped kinase inhibitor in Neospora caninum and Danio rerio. Int. J. Mol. Sci. 23, 2381. doi: 10.3390/ijms23042381. PMID: 35216497 PMC8879773

[B48] Murphy R. C. Ojo K. K. Larson E. T. Castellanos-Gonzalez A. Perera B. G. K. Keyloun K. R. . (2010). Discovery of potent and selective inhibitors of CDPK1 from C. parvum and T. gondii. ACS Med. Chem. Lett. 1, 331–335. doi: 10.1021/ml100096t. PMID: 21116453 PMC2992447

[B49] Neville A. J. Zach S. J. Wang X. Larson J. J. Judge A. K. Davis L. A. . (2015). Clinically available medicines demonstrating anti-Toxoplasma activity. Antimicrob. Agents Chemother. 59, 7161–7169. doi: 10.1128/AAC.02009-15. PMID: 26392504 PMC4649158

[B50] Ojo K. K. Larson E. T. Keyloun K. R. Castaneda L. J. DeRocher A. E. Inampudi K. K. . (2010). Toxoplasma gondii calcium-dependent protein kinase 1 is a target for selective kinase inhibitors. Nat. Struct. Mol. Biol. 17, 602–607. doi: 10.1038/nsmb.1818. PMID: 20436472 PMC2896873

[B51] Paquet C. Yudin M. H. Allen V. M. Bouchard C. Boucher M. Caddy S. . (2013). Toxoplasmosis in pregnancy: prevention, screening, and treatment. J. Obstet. Gynaecol. Can. 35, 78–81. doi: 10.1016/s1701-2163(15)31053-7. PMID: 23343802

[B52] Regidor-Cerrillo J. Largo de la Torre A. Sánchez-Sánchez R. Ferre I. Moreno-Gonzalo J. Ortega-Mora L. M. (2026). A safe and efficacious inactivated vaccine aids prevent reproductive failure associated to congenital toxoplasmosis in ovine. Vet. Res. 57, 40. doi: 10.1186/s13567-026-01720-2. PMID: 41857611 PMC13003711

[B53] Robert-Gangneux F. (2014). It is not only the cat that did it: how to prevent and treat congenital toxoplasmosis. J. Infect. 68, S125–S133. doi: 10.1016/j.jinf.2013.09.023. PMID: 24119928

[B54] Sánchez-Sánchez R. Ferre I. Regidor-Cerrillo J. Gutiérrez-Expósito D. Ferrer L. M. Arteche-Villasol N. . (2019). Virulence in mice of a Toxoplasma gondii type II isolate does not correlate with the outcome of experimental infection in pregnant sheep. Front. Cell. Infect. Microbiol. 8. doi: 10.3389/fcimb.2018.00436. PMID: 30662874 PMC6328472

[B55] Sánchez-Sánchez R. Huertas-López A. Largo-de la Torre A. Ferre I. Dini F. M. Re M. . (2025). Treatment with BKI-1748 after Toxoplasma gondii systemic dissemination in experimentally infected pregnant sheep improves foetal and lamb mortality and morbidity and prevents congenital infection. Antimicrob. Agents Chemother. 69, 1448. doi: 10.1128/aac.01448-24. PMID: 39745365 PMC11823607

[B56] Sánchez-Sánchez R. Imhof D. Hecker Y. P. Ferre I. Re M. Moreno-Gonzalo J. . (2024). An early treatment with BKI-1748 exhibits full protection against abortion and congenital infection in sheep experimentally infected with Toxoplasma gondii. J. Infect. Dis. 229, 558–566. doi: 10.1093/infdis/jiad470. PMID: 37889572 PMC10873186

[B57] Sanchez-Sanchez R. Vazquez P. Ferre I. Ortega-Mora L. M. (2018). Treatment of toxoplasmosis and neosporosis in farm ruminants: state of knowledge and future trends. Curr. Top. Med. Chem. 18, 1304–1323. doi: 10.2174/1568026618666181002113617. PMID: 30277158 PMC6340160

[B58] Shammaa A. M. Powell T. G. Benmerzouga I. (2021). Adverse outcomes associated with the treatment of Toxoplasma infections. Sci. Rep. 11, 1035. doi: 10.1038/s41598-020-80569-7. PMID: 33441899 PMC7806722

[B59] Stelzer S. Basso W. Silván J. B. Ortega-Mora L. M. Maksimov P. Gethmann J. . (2019). Toxoplasma gondii infection and toxoplasmosis in farm animals: Risk factors and economic impact. Food Waterborne Parasitol. 15, 37. doi: 10.1016/j.fawpar.2019.e00037. PMID: 32095611 PMC7033994

[B60] Thiébaut R. Leproust S. Chêne G. Gilbert R. (2007). Effectiveness of prenatal treatment for congenital toxoplasmosis: a meta-analysis of individual patients’ data. Lancet 369, 115–122. doi: 10.1016/S0140-6736(07)60072-5. PMID: 17223474

[B61] Torgerson P. R. Mastroiacovo P. (2013). The global burden of congenital toxoplasmosis: a systematic review. Bull. World Health Organ. 91, 501–508. doi: 10.2471/BLT.12.111732. PMID: 23825877 PMC3699792

[B62] Trees A. J. Crozier S. J. Buxton D. Blewett D. A. (1989). Serodiagnosis of ovine toxoplasmosis: an assessment of the latex agglutination test and the value of IgM specific titres after experimental oocyst-induced infections. Res. Vet. Sci. 46, 67–72. doi: 10.1016/s0034-5288(18)31120-2 2646662

[B63] Vallejo R. Benavides J. Arteche-Villasol N. Sánchez-Sánchez R. Calero-Bernal R. Ferreras M. C. . (2023). Experimental infection of sheep at mid-pregnancy with archetypal type II and type III Toxoplasma gondii isolates exhibited different phenotypic traits. Vet. Parasitol. 315, 109889. doi: 10.1016/j.vetpar.2023.109889. PMID: 36753878

[B64] Van Voorhis W. C. Doggett J. S. Parsons M. Hulverson M. A. Choi R. Arnold S. . (2017). Extended-spectrum antiprotozoal bumped kinase inhibitors: A review. Exp. Parasitol. 180, 71–83. doi: 10.1016/j.exppara.2017.01.001. PMID: 28065755 PMC5498274

[B65] Vargas-Villavicencio J. A. Besné-Mérida A. Correa D. (2016). Vertical transmission and foetal damage in animal models of congenital toxoplasmosis: A systematic review. Vet. Parasitol. 223, 195–204. doi: 10.1016/j.vetpar.2016.04.024. PMID: 27198800

[B66] Vargas-Villavicencio J. A. Cañedo-Solares I. Correa D. (2022). Anti-Toxoplasma gondii IgM long persistence: what are the underlying mechanisms? Microorganisms 10, 1659. doi: 10.3390/microorganisms10081659. PMID: 36014077 PMC9415799

[B67] Verhelst D. De Craeye S. Entrican G. Dorny P. Cox E. (2014). Parasite distribution and associated immune response during the acute phase of Toxoplasma gondii infection in sheep. BMC Vet. Res. 10, 293–295. doi: 10.1186/s12917-014-0293-5. PMID: 25511864 PMC4279818

[B68] Villard O. Cimon B. L’Ollivier C. Fricker-Hidalgo H. Godineau N. Houze S. . (2016). Serological diagnosis of Toxoplasma gondii infection: recommendations from the French National Reference Center for Toxoplasmosis. Diagn. Microbiol. Infect. Dis. 84, 22–33. doi: 10.1016/j.diagmicrobio.2015.09.009. PMID: 26458281

[B69] Wallace J. M. Shepherd P. O. Milne J. S. Aitken R. P. (2021). Perinatal complications and maximising lamb survival in an adolescent paradigm characterised by premature delivery and low birthweight. PloS One 16, e0259890. doi: 10.1371/journal.pone.0259890. PMID: 34780509 PMC8592415

[B70] Wallon M. Garweg J. G. Abrahamowicz M. Cornu C. Vinault S. Quantin C. . (2014). Ophthalmic outcomes of congenital toxoplasmosis followed until adolescence. Pediatrics 133, e601–e608. doi: 10.1542/peds.2013-2153. PMID: 24534412

[B71] Withoeft J. A. Marian L. da Costa L. S. Fernandes F. D. Vogel F. S. F. das Neves G. B. . (2024). Sheep abortions associated with Neospora caninum and Toxoplasma gondii infections in multiple flocks from Southern Brazil. Vet. Res. Commun. 48, 2699–2705. doi: 10.1007/s11259-024-10390-4. PMID: 38653939

