# Supplementary material for: BKI-1748 confers a high level of protection against ovine congenital toxoplasmosis when administered after IgM seroconversion
Source: Front Cell Infect Microbiol. 2026 Apr 27;16:1819490. doi: 10.3389/fcimb.2026.1819490 (PMC13158197; doi:10.3389/fcimb.2026.1819490)
Supplement: Supplementary File 1 — Rectal temperatures (A) and serum anti-T. gondii-IgM levels (B) during the first two weeks post-infection. [file Table1.docx]

**A**


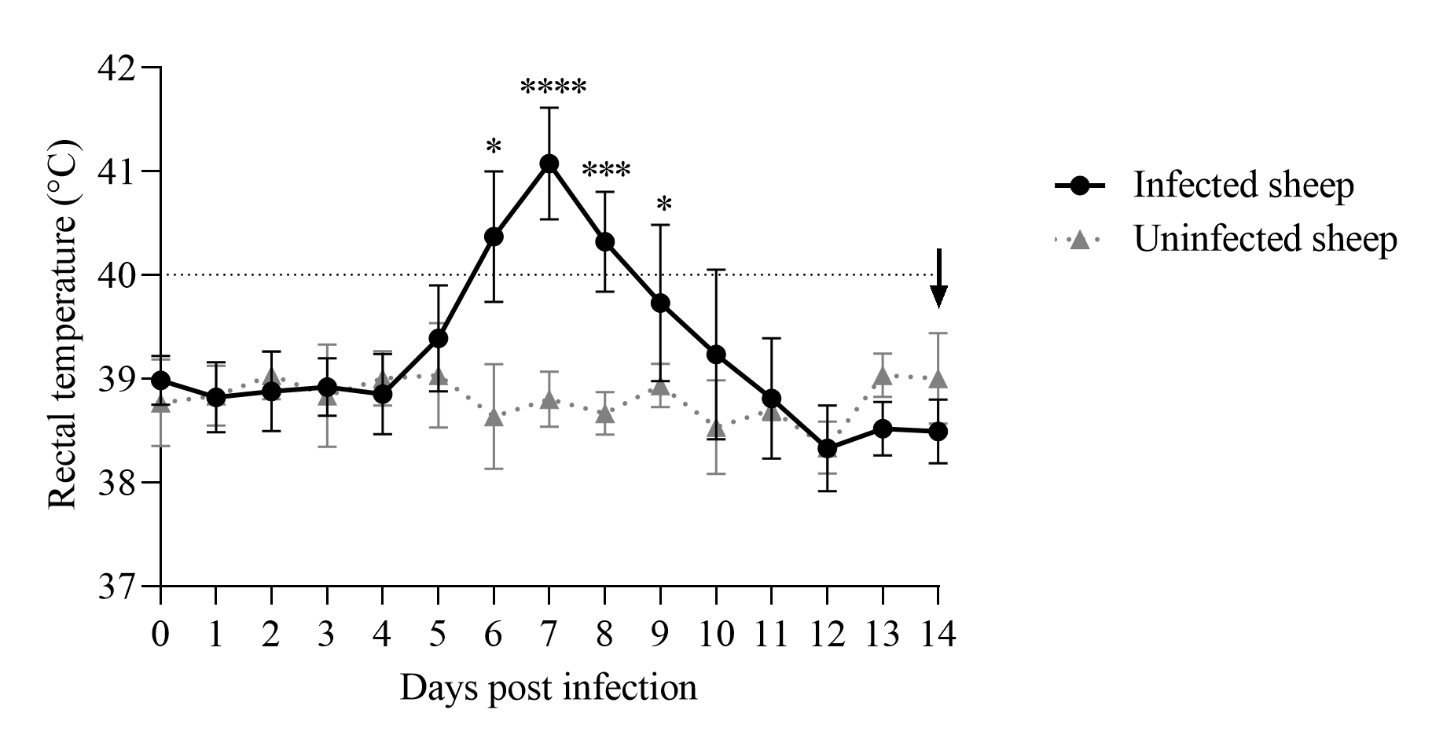


Each point represents the mean + SD for infected or uninfected sheep. Horizontal dashed line indicates the upper threshold for physiological rectal temperature in sheep, and the black arrow points the beginning of the treatment. For significant differences between infected and non-infected sheep, (*) indicates P < 0.05, (***) indicates P < 0.001 and (****) indicates P < 0.0001.

**B**


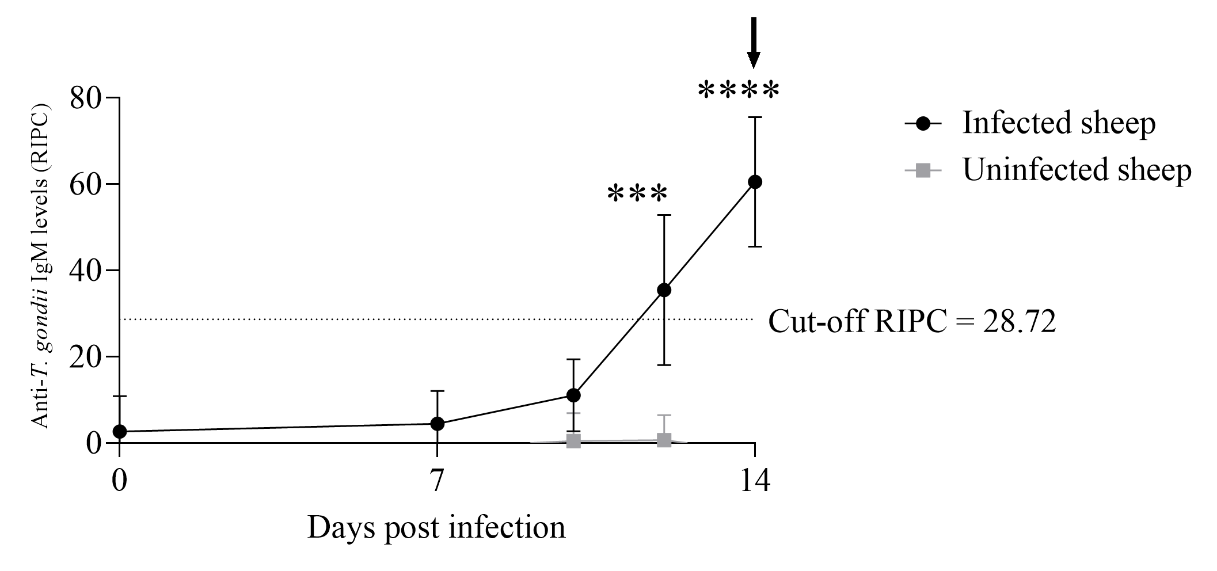


Anti-*T. gondii* IgM levels are expressed in relative index percent (RIPC). Each point represents the mean + SD for infected or uninfected sheep. The black arrow points at the time point of treatment start. For significant differences between infected and non-infected sheep, (***) indicates P < 0.001 and (****) indicates P < 0.0001.
