# Supplementary material for: BKI-1748 confers a high level of protection against ovine congenital toxoplasmosis when administered after IgM seroconversion
Source: Front Cell Infect Microbiol. 2026 Apr 27;16:1819490. doi: 10.3389/fcimb.2026.1819490 (PMC13158197; doi:10.3389/fcimb.2026.1819490)
Supplement: Supplementary File 2 — Plasma levels of BKI-1748 in infected and treated dams. [file Table2.docx]

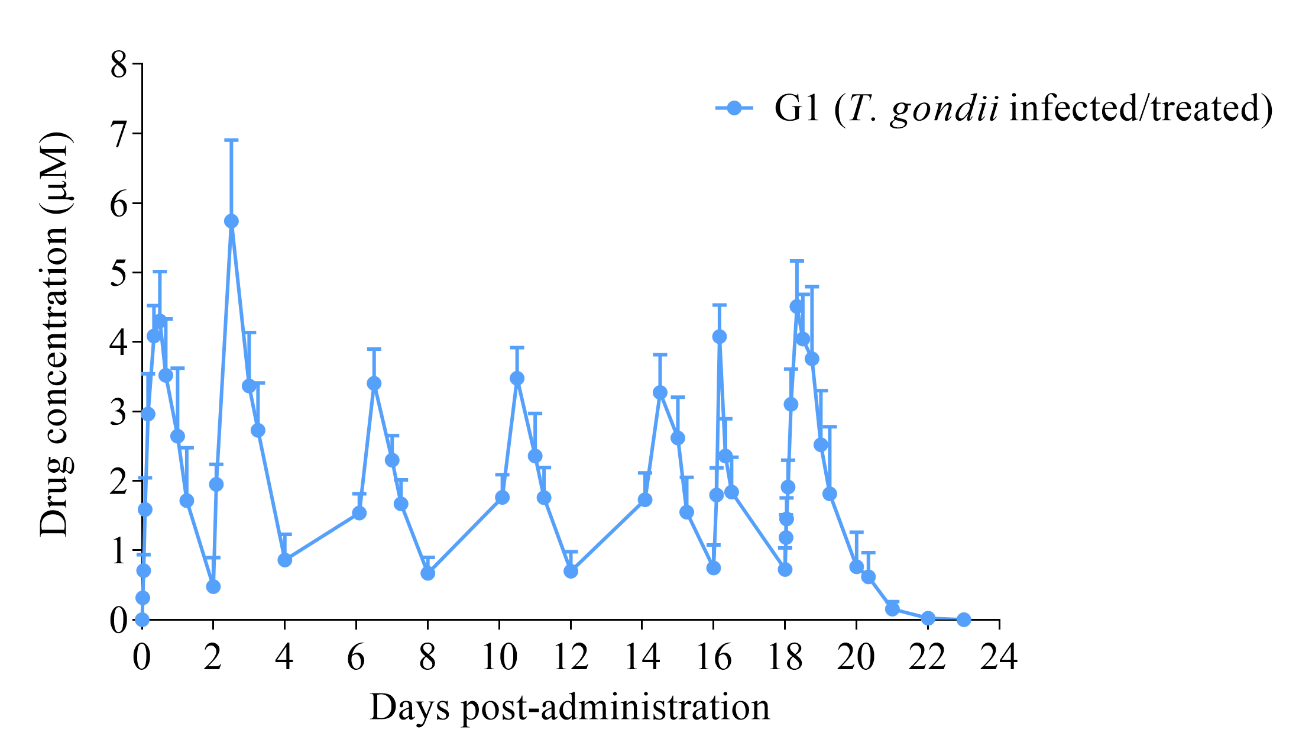


An oral dose of BKI-1748 at 15 mg/kg was administered, starting on day 14 p.i, every two days for a total of up to ten treatments. The mean concentrations with standard deviations are shown for each sampling time.
