# Supplementary material for: BKI-1748 confers a high level of protection against ovine congenital toxoplasmosis when administered after IgM seroconversion
Source: Front Cell Infect Microbiol. 2026 Apr 27;16:1819490. doi: 10.3389/fcimb.2026.1819490 (PMC13158197; doi:10.3389/fcimb.2026.1819490)
Supplement: Supplementary File 4 — Parasite loads in tissues from fetuses/lambs in the infected/untreated group (G2). [file Table4.docx]

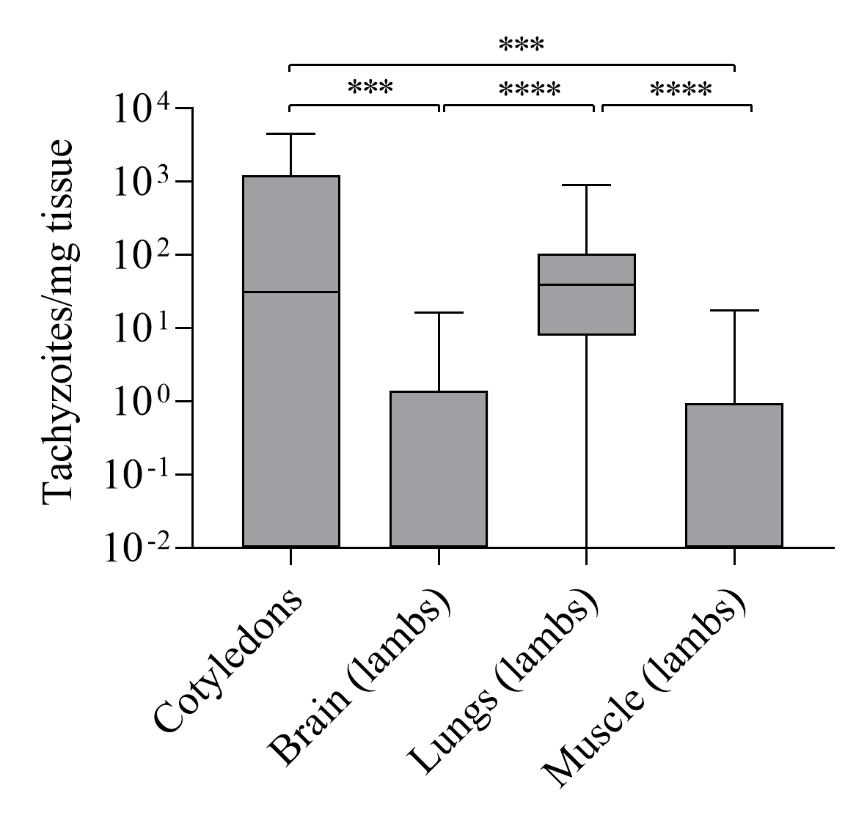


For significant differences, (***) indicates P < 0.001 and (****) indicates P < 0.0001. As the detection limit for *T. gondii* by real-time PCR was 0.1 parasites, negative samples (0 parasites) were plotted on a logarithmic scale as < 0.1 (i.e., 10^-2^).
