# Supplementary material for: BKI-1748 confers a high level of protection against ovine congenital toxoplasmosis when administered after IgM seroconversion
Source: Front Cell Infect Microbiol. 2026 Apr 27;16:1819490. doi: 10.3389/fcimb.2026.1819490 (PMC13158197; doi:10.3389/fcimb.2026.1819490)
Supplement: Supplementary File 5 — Box plots illustrating parasite burdens in fetal/lamb tissues and placental cotyledons. [file Table5.docx]

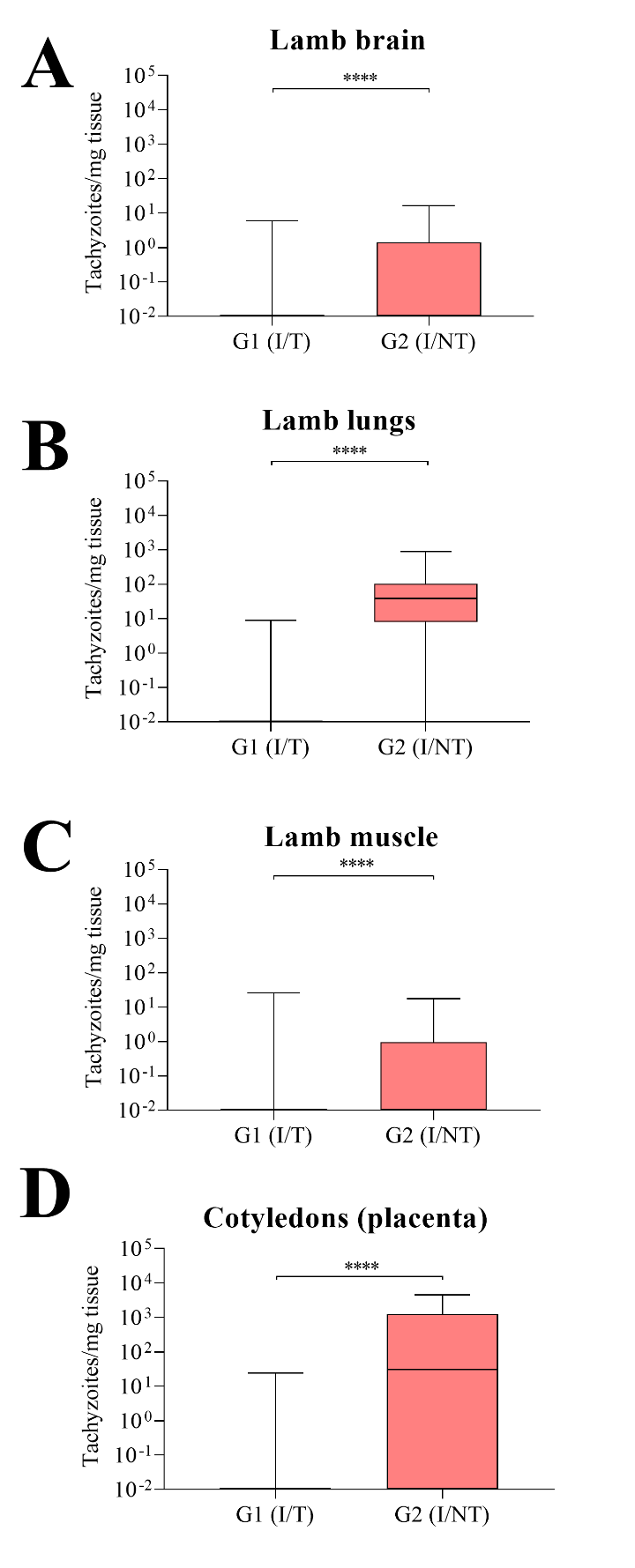


Parasite burden in brain (A), lungs (B), and semitendinosus muscle (C) of foetuses/lambs and placental cotyledons (D). The graphs display the median percentage, interquartile range (boxes), and minimum and maximum values (whiskers) for each organ in groups G1 (I/T) and G2 (I/NT). For significant differences, (****) indicates P < 0.0001. As the detection limit for *T. gondii* by real-time PCR was 0.1 parasites, negative samples (0 parasites) were plotted on a logarithmic scale as < 0.1 (i.e., 10^-2^).
